# Anglo-Swiss Milk Food

**Published:** 1882-02-20

**Authors:** 


WE invite attention to the advertisement in this issue of the Anglo-Swiss Milk Food for infants and invalids. This preparation has attained to high reputation and practitioners who so often need such articles in practice would do well to give it a trial.

